# Contributing Factors of Presenteeism among Portuguese and Swiss Nurses: A Qualitative Study Using Focus Groups

**DOI:** 10.3390/ijerph19148844

**Published:** 2022-07-21

**Authors:** Carlos Laranjeira, Filipa Pereira, Ana Querido, Marion Bieri, Henk Verloo

**Affiliations:** 1 School of Health Sciences, Polytechnic of Leiria, Campus 2, Morro do Lena, Alto do Vieiro, Apartado 4137, 2411-901 Leiria, Portugal; ana.querido@ipleiria.pt; 2 Centre for Innovative Care and Health Technology (ciTechCare), Polytechnic of Leiria, Campus 5, Rua de Santo André—66–68, 2410-541 Leiria, Portugal; 3 Research in Education and Community Intervention (RECI I&D), Piaget Institute, 3515-776 Viseu, Portugal; 4 School of Health Sciences, HES-SO Valais/Wallis, 5 Chemin de l’Agasse, CH-1950 Sion, Switzerland; filipa.pereira@hevs.ch (F.P.); marionbieri21@gmail.com (M.B.); henk.verloo@hevs.ch (H.V.); 5 Center for Health Technology and Services Research (CINTESIS), NursID, University of Porto, 4200-450 Porto, Portugal; 6 Service of Old Age Psychiatry, Lausanne University Hospital, Route de Cery 60, CH-1008 Prilly, Switzerland

**Keywords:** presenteeism, focus groups, qualitative study, causes, nurses’ perceptions, quality of care, healthcare settings

## Abstract

Evidence of nurse presenteeism has mainly focused on quantifying its prevalence and consequences on productivity, quality of care, and patient safety. Few data exist on nurses’ perceptions of their presenteeism and its related causes. We explored concepts of presenteeism and its contributing factors with frontline nurses and nurse managers in different healthcare settings in Portugal and Switzerland. Our qualitative study design involved 8 online focus groups involving 55 participants. The transcribed data was explored using thematic analysis. Three main reasons for presenteeism were identified: unfamiliar terminology; the paradoxical effect of `being present’ but absent; and presenteeism as a survival strategy. Six contributing factors were also recognized: (a) institutional disinterest toward employees; (b) paradigm shift: the tension between person-centered and task-centered care; (c) sudden changes in care practices due to the COVID-19 pandemic; (d) a lack of shared work perspectives with hierarchical superiors; (e) the financial burden of being absent from work; and (f) misfit of human responses. This study generates valuable, in-depth knowledge about the concepts and causes of presenteeism, and significant suggestions for the broader audience of nurse managers and leaders seeking to improve the quality of care.

## 1. Introduction

Healthcare professionals, especially nurses, are often exposed to “heavy workloads, shift work, and irreplaceable duties”. They nevertheless “continue working despite feeling unwell”, a phenomenon called *presenteeism* juxtaposed with the phenomenon of absenteeism [1] (p. 1).

In the last decade, the majority of acute, long-term, and community healthcare settings have been increasingly challenged by human resources issues and crises due to staff shortages, especially among qualified professional staff such as nurses and allied healthcare professionals. High workloads, complex care regimes, and elevated staff turnover or shortages can lead to consequences such as absenteeism and presenteeism that can influence the quality and safety of patient care [2]. Presenteeism and absenteeism are closely related phenomena as “both encompass disease conditions with variable personal, biological, environmental, family, financial or functional causes” [3] (p. 98). The literature indicates “that presenteeism increases medical costs, reduces productivity, elevates the rate of work accidents, and causes financial losses to organizations” [3] (p. 98). An awareness of presenteeism is critical for nurses’ occupational health and nursing personnel management. Furthermore, the literature reveals that the organizational context plays an important role in presenteeism, and a supportive working environment tends to reduce the trends toward presenteeism [4].

One commonly used definition of presenteeism in nursing is the “act of being physically present at work when one should not be there” [5] (p. 620). Presenteeism is a multifaceted concept influenced by a variety of individual (e.g., personality traits and career development stages), organizational (e.g., work environment, work schedule, and job content), and contextual factors (e.g., welfare and social security systems, work regulations, replaceability, and rewards) [6,7].

Historically, studies on presenteeism have concentrated on two perspectives—North American and European—each with their own study paradigms and particular contextual and social system conditions [8]. North American major strategy focuses were on productivity losses at work due to presenteeism, whereas European focuses typically examined the lack of staff job security and dangers to their future health. Combining these two different perspectives on presenteeism is critical to a deeper understanding of its intricacies [9]. Some of the literature suggests that presenteeism is a personal decision; however, one cross-cultural study on presenteeism found significant disparities in attendance behavior between the nations examined [10]. Basic cultural differences may influence what is considered a legitimate cause for absence or presence, and countries differ in their national norms and regulations and how they respond to presenteeism [10]. 

Nurse presenteeism has long been of international concern, with impacts on nurse staffing levels, patient care, and hospital costs [11,12,13]. Nursing has been identified as one of the professions with the highest rates of presenteeism [14,15,16]. In a systematic review by Webster et al. [17] (p. 4), “the overall presenteeism prevalence ranged from 35 to 97%, and for studies of participants who worked in the healthcare sector, this was 37 to 97%”. The variability of prevalence rates should be explained considering the following elements: (a) sample characteristics (population, response rate), (b) the types of presenteeism studied (sickness, non-sickness, overall), and (c) the variety of measurement instruments [17]. A recent meta-analysis of 28 studies from 14 countries confirmed that presenteeism is prevalent (estimated at around 49%) among the nursing workforce [18]. This was “attributable to multiple health conditions and stress; and that it is tied to multiple consequences for the economy, patient safety, and nurse well-being” [19] (p. 9).

Sickness (attending work when sick) and job stress (when work stress affects performance) have both been linked to nurse presenteeism [5]. Presenteeism has a detrimental influence on nurses’ physical and mental health, diminishes job satisfaction and engagement, and promotes job burnout; however, it also affects the medical institutions’ income and productivity [16,20,21,22,23]. Most prior research on nurse presenteeism has focused on measuring the prevalence and consequences of presenteeism [13,17,20,24]. However, nurses have seldom been invited to clarify their own definitions of “being presentee” and identify any underlying causes. Their responses provide information for future studies and on areas requiring intervention. Moreover, perceptions of presenteeism are determined by social settings, including the healthcare institution’s managerial, structural, and organizational culture [11,12]. 

The global health crisis caused by the COVID-19 pandemic has fostered a greater interest in and a need to understand nurse presenteeism [25,26]. According to the International Council of Nursing, nurses were the frontline workers most affected by the pandemic [27]. During this crisis, nurses cared for patients who exhibited not only new or worsening health problems but also significant levels of anxiety and distress [28]. 

To the best of our knowledge, there has been little research on individuals’ conceptions of presenteeism based on their lived experiences. We thus chose a qualitative methodology to explore and understand nurse presenteeism, as it is hard to explain this phenomenon using simple external factors alone. Moreover, little is known about the contextual situations, processes, or motivations of nurses who provide care while they are sick [29]. Furthermore, few studies have explored presenteeism across multiple sites and from an international perspective. The present study, therefore, focused on different types of care settings (acute and long-term) and different professional positions (frontline nurses and nurse managers).

Over the last 10 years, there has been a significant migration of nurses from southern European countries toward western European ones, notably from Portugal to Switzerland [30]. The present study investigated the perceptions and experiences of nurses in both countries. While working conditions tend to be more attractive in Switzerland, both countries face similar work-related issues: a deficit of human resources, physical and psychological strains, job insecurity, and high turnover rates [31,32]. 

This study’s main purpose, therefore, was to explore the factors associated with presenteeism among frontline nurses and nurse managers in acute, primary, and long-term healthcare settings in Portugal and Switzerland. The study’s research questions were: (a) How do nurses perceive the concept of presenteeism? and (b) What do nurses perceive to be the causes of presenteeism? 

## 2. Materials and Methods

### 2.1. Study Design

This study is part of a larger international project to study the link between presenteeism in the nursing workforce and its impact on the quality and safety of patient care [33]. 

We conducted a qualitative descriptive study involving online focus groups (FGs) and designed with regard to Krueger and Casey’s [34] methodological framework. Group participation in FGs enables the in-depth study of personal perspectives and provides facts that would be hard to gather using other approaches [35]. Group interactions can help screen out extreme or misguided viewpoints, improving data quality [34]. Effective FGs cover a range of relevant themes, with specificity and depth, and focus on participant experiences and the interplay between those experiences [35]. This study is described following the COnsolidated criteria for REporting Qualitative research (COREQ) checklist [36].

### 2.2. Sample Selection and Recruitment

The FGs involved 55 participants and took place over 5 months (from March 2021 to July 2021). The Portuguese research team conducted four online FGs in acute care hospital settings: two with frontline nurses active in acute care wards (e.g., emergency, internal medicine, and surgery) and two with nurse managers (with frontline clinical leadership responsibilities at the unit level). The Swiss research team conducted four online FGs: two in long-term residential care facilities and two in community healthcare settings, all with frontline nurses and nurse managers [33]. 

Participants were recruited using a purposive sampling technique. The inclusion criteria were: (a) working in a public or private healthcare setting with at least 1 month’s experience in their current workplace (which is officially considered the time necessary for integration); (b) working at least 20% of a full-time equivalent position; and (c) having a bachelor’s, master’s, or a PhD degree. It was important for us, knowing the clinical reality and organizational constraints, to impose the inclusion criteria of participants having had at least 1 month’s experience in their current workplace. This would give them the time to integrate and to be able to express themselves about the workload and organizational culture and procedures. Participants also had to be able to connect to the internet using a suitable device. Prior to the FGs, the lead local researcher contacted the participants by email or telephone. As presenteeism affects not only presentees but also those who work with them, we did not exclude nurses who had never worked while feeling unwell or unable to.

We sought a balanced number of nurses in each FG to ensure relative homogeneity but enough variety to allow for differing viewpoints. To acquire different data on nurse experiences of presenteeism, no restrictions were placed on their gender, age, or seniority. This study’s rationale and interest were explained to participants upon invitation to engage in the FG.

### 2.3. Data Collection

We conducted eight FGs (four in each country) with frontline nurses and nurse managers, gathering a broad range of opinions, attitudes, and experiences from diverse sociocultural contexts. Each 1.5- to 2-h FG was facilitated by 2 researchers with significant relevant knowledge of the national and local setting. The first, an experienced research nurse, moderated the meeting, explained its aims, and encouraged the exchange of ideas. Meanwhile, the second observed the atmosphere, interactions, and conversation flow, and recorded field notes. Real-time data of the interactions between participants and the researchers was gathered using videoconferencing technology [37,38].

Each of the eight FGs started with participants completing a data sheet with questions about their age, sex, work function, years of experience as a nurse, and their current work area. The researchers then presented a situationally and culturally adapted vignette about presenteeism [33]. Indeed, the vignette’s descriptions of real-life circumstances impressed upon the participants that meanings are social and situational. The authors developed the interview guide based on a literature analysis [5] and their practical expertise and experiences of presenteeism. A pilot FG was held prior to our planned data collection to test the interview guide’s questions and ensure smooth interaction between the moderator and the observer. Because only minimal changes were made to the interview guide, data from the pilot FG was also used in our final analysis. The vignette presented a hypothetical situation demanding action or judgment from the participants, followed by the open question: “Based on the vignette, what is presenteeism, and what are its contributing factors?” The research team then asked more specific questions based on the responses to the first question, such as: (a) “How do you view presenteeism among nursing staff?”; (b) “Why do nurses find it helpful to remain at work or return to work when they have health problems?”; (c) “What are the main reasons for this?”; (d) “Could you describe any situations where you were at work while unwell?”; and (e) “If you have worked while ill, how did you manage it?”

The two most important factors in cross-national FG research are that questions address the same topics in each country and that all the participants understand the questions in exactly the same way.

### 2.4. Ethics

At the beginning of each FG, participants were ensured of full confidentiality of the results and asked whether they agreed to either audio or video recording of the sessions. Participants were also informed about the professional backgrounds of the moderator and observer, the rationale for their interest in the issue, and that FG data would be analyzed and submitted for publication. All research participants gave their permission to be part of the study and were asked to provide written informed consent. Volunteers received no compensation for their participation. All study data were kept in a padlocked drawer, and digital data (such as audio recordings and transcripts) were kept on a password-protected desktop computer.

### 2.5. Data Analysis

The research team used Braun and Clarke’s six-step guide for thematic analysis to analyze the transcribed interview data (Table 1). 

Thematic analysis is a useful tool for assessing diverse participant views, showing commonalities and contrasts, and uncovering unexpected findings. This method is also useful for processing data in a well-structured manner, resulting in a clear, well-organized final report [39]. Data analysis began immediately after each meeting and continued until data saturation was achieved.

First, group discussions were fully transcribed and analyzed to ensure fidelity to the original meaning. The researchers’ notes contributed to interpreting certain aspects of the discussion. All the researchers read the transcriptions thoroughly, allowing them to become familiar with the data and obtain an overview. Next, initial codes were generated deductively, based on prior research and our conceptual framework [40], and potential themes were sought using WebQDA^®^. Codes were adjusted to fit two pre-existing frameworks containing aspects of interest, specifically those provided by Rainbow and Steeg [5] (who proposed a conceptual model of presenteeism in nursing, and the underlying links between presenteeism’s antecedents, attributes, and consequences) and by Pit and Hansen [41] (who proposed elements that precipitate presenteeism). 

A branching hierarchical tree was created to search for themes and examine them in connection with both coded extracts and the complete dataset. To settle upon the initial sub-themes, the authors met many times, discussed the coding process, and collaborated on naming common themes and sub-themes. The data from the two countries was combined and analyzed. Each theme and sub-theme were summarized, and excerpts exemplifying their essence were chosen. To ensure that quotations were accurately translated, they were first translated into English and then backtranslated into Portuguese and French. Example extracts from the participants were numbered according to the FG number (1–8), the country (PT/CH), and the nurse’s function (frontline/manager).

### 2.6. Research Rigor

The strategies to ensure data trustworthiness, dependability, and credibility were carried out through member checking, researcher triangulation with peer-debriefing, reflexivity, and an audit trail [40]. 

To ensure trustworthiness, each participant received a transcript of their coded FG interview and was asked to confirm that the codes matched their own experiences. However, no feedback on the findings was provided. To ensure fittingness and audibility, three independent researchers analyzed the transcripts and checked that respondents indicated the probable causes of presenteeism among nurses. This also reinforced trustworthiness [40]. The research team attempted to address reflexivity during the study’s design phase. This team included one psychologist and four registered nurses, all of whom had significant clinical experience and had previously conducted studies employing qualitative methods. Before beginning the research, the researchers discussed their understanding of the project’s purpose and methods and later participated in data analysis.

## 3. Findings

### 3.1. Sample Description

Participants included 55 nurses: 48 females (87.3%) and 7 males (12.7%). Their mean age was 45.03 (SD = 8.47) years, ranging from 25 to 61 years. The average work experience was 19.78 years, ranging from 3 to 40 years. Regarding healthcare settings, most participants (70.9%) worked in acute hospitals and 20.1% in primary and long-term care settings. Regarding educational level, 26 (47.3%) had a bachelor’s degree, 28 (50.9%) had a master’s degree, and none had a PhD (Table 2).

### 3.2. Findings from the FGs

**Figure 1:**
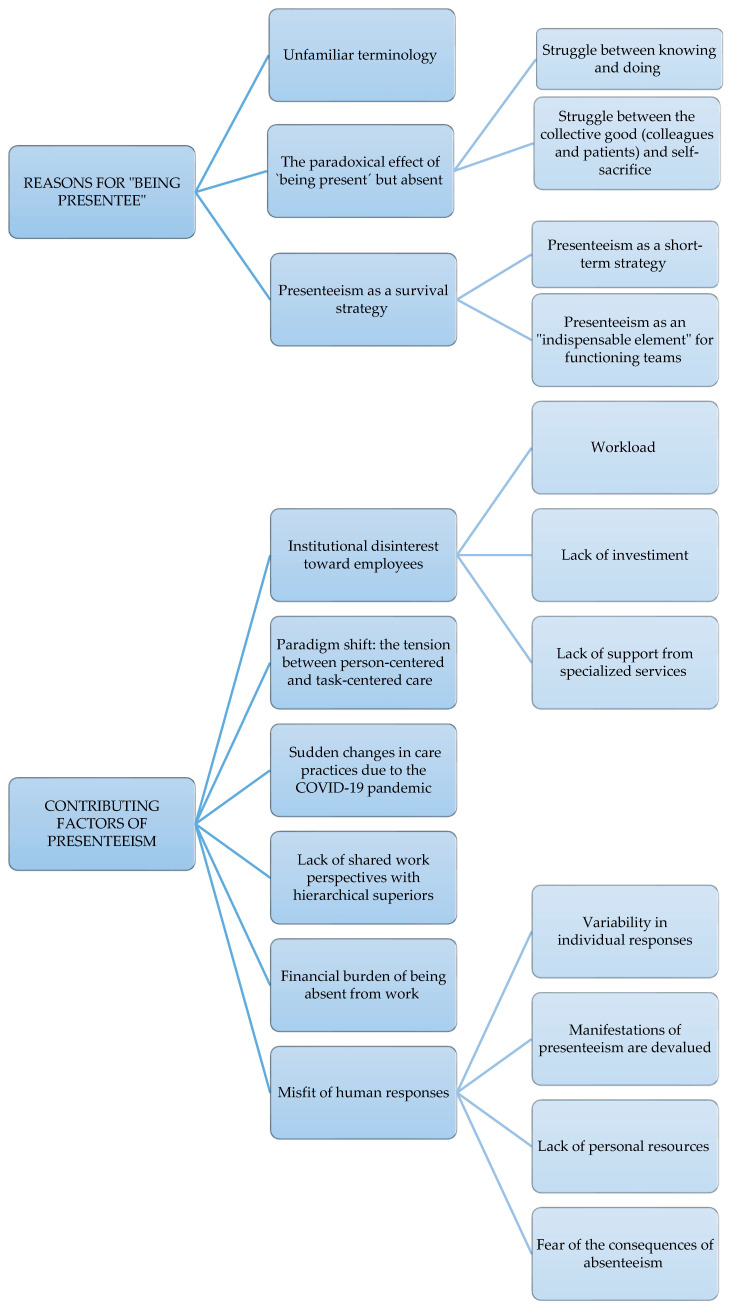
Key themes and sub-themes illuminated from thematic data analysis of nurses’ perceptions and experiences of presenteeism.

In total, 2 overarching themes, 9 themes, and 11 sub-themes were extracted from the data on nurses’ perceptions and experiences related to presenteeism (see Figure 1).

#### 3.2.1. Reasons for “Being Presentee”

Nurses invoked multiple reasons for being present at work while feeling unwell, i.e., their presenteeism. In all the FGs, these factors included: “unfamiliar terminology”, “the paradoxical effect of ’being present’ but absent”, and “presenteeism as a survival strategy”. 

A lack of knowledge about presenteeism contributed to the inability to recognize that behavior as problematic and reportable. For many participants, presenteeism was an unfamiliar phenomenon, whose complexity was not always understood by frontline nurses or nurse managers:

*“When I was invited, the theme (...) the nomenclature was familiar to me, but I had some difficulty perceiving the context, the evidence that already existed... for presentism”. (FG3–PT-nurse managers)* *“It’s true that I only became aware of this term, of this swear word, when I received the flyer to participate in the study”. (FG6-CH-frontline nurses)*

Despite unfamiliarity with the term “presenteeism”, participants mentioned that coming to work when ill or injured is a common practice. Nurses felt obligated to be at work. They experienced the paradoxical effect of being present but also absent, which was discernible in a struggle between knowing and doing. In other words, the knowing–doing gap leads nurses to resort to being a presentee at work:

*“So, there’s always a bit of a paradox, and well, we’re carers! We give our patients a lot of advice, all the time, about well-being, the importance of taking care of oneself, etc. We harp on about this all day long and then, finally, we are not able to apply it to ourselves”. (FG7-CH-frontline nurses)*

There is an implicit belief in many healthcare institutions that deciding on action is equivalent to taking action to implement that decision. This can actually become a barrier to tangible action:

*“There’s always this gap where we don’t apply it; we don’t apply well-being to ourselves”. (FG7-CH-frontline nurses)*

Working at one’s full potential was a recurring theme among FG participants. Furthermore, some nurses noted that they attended work (perhaps more than they should) for other reasons, including because they were willing to sacrifice themselves for the collective good (colleagues and patients). Teamwork was understood to imply a readiness to strive, by taking on a heavy workload, for the benefit of co-workers and patients. Some participants explained they felt compelled to be a presentee:

*“It’s not sentimentality; it’s a kind of loyalty to my colleagues” (FG7-CH-frontline nurses); “Nursing is always being present, because our colleagues and patients need us…” (FG2-PT-frontline nurses)*

Interviewees frequently described “being presentee” as a short-term survival strategy:

*“(...) in a way, it is my survival too! As I said, it’s a coping strategy. Because it is the least-bad choice. (...) In the short term, exactly. Because in the long term, it usually comes back to hit us. But we all work in the same way. In general, we always choose what’s positive in the short term. It’s the same”. (FG7-CH-frontline nurses)*

Overall, nurses appeared available to work themselves to exhaustion and even incapacitation:

*“We run ourselves to the very end, until you just can’t deal with it anymore”. (FG2-PT-frontline nurses)*

Survival was also seen as an “indispensable element” for team functioning:

*“I think it’s even more [indispensable] for a manager. Sometimes presenteeism is essential. Yes, yes, we also replace [each other], we fill the gaps when there are absences, we go, we work. No, but it’s, there you go, we all do overtime (...)”. (FG4-CH-nurse managers)*

#### 3.2.2. Contributing Factors of Presenteeism

Respondents mentioned a wide range of events and reasons for presenteeism, which were grouped into six contributing factors: (a) institutional disinterest toward employees; (b) paradigm shift: the tension between person-centered and task-centered care; (c) sudden changes in care practices due to the COVID-19 pandemic; (d) lack of shared work perspectives with hierarchical superiors; (e) financial burden of being absent from work; and (f) misfit of human responses.

(a) Institutional disinterest toward employees

Participants stated that professional overload made it difficult for nurses to care for others and posed a danger to workers themselves. This scenario can create a vicious cycle, whereby healthy nurses become sick or are injured in the workplace yet are then expected to maintain their existing workloads in that condition. The lack of a supportive environment may cause job dissatisfaction and reduce employee trust in the organization. On this point, participants stated:

*“The boss forgets that we are human too!” (FG2-PT-frontline nurses); “The concept of presenteeism is related to professionals’ excessive workloads and the lack of responses to the professionals’ needs”. (FG1-PT-frontline nurses)*

Some nurse managers adopted a “sandwich” metaphor because, as middle-level managers, they support upper management by implementing organizational policies and decisions, and they support their subordinates, particularly when it comes to policies relating to the delivery of care:

*“I feel sandwiched because I have to obey those above me and provide results, but I can also understand what… the bottom of the sandwich is suffering and that they have difficulties and needs, and sometimes I don’t have the tools to do all that…” (FG4-PT–nurse managers)*

Nurses also pointed out that they sometimes felt abandoned by managers and leaders. Nurses, it appeared, overwhelmingly requested better assistance to do their tasks more efficiently. They frequently expressed concerns about a lack of organized or appropriate help, and they desired improved management support mechanisms:

*“I relate it to institutional disinvestment in the wealth represented by human resources, in how to keep people motivated”; “What matters is having X staff working that day. Their condition doesn’t matter; the context they’re in doesn’t matter; whether they are properly emotionally or technically prepared, it doesn’t matter…” (FG1-PT-frontline nurses)*

Support is a cornerstone of management practice, and it is critical for employees to feel engaged in an organization. However, some participants discussed the lack of support from specialized (occupational) services:

*“Yes, there is a lack of answers... The few answers that exist are difficult to access [specialized services]. There is some stigma for any colleagues who seek them out. They exist, but they are not well used!”; “There is a lack of answers from institutions”. (FG1-PT-frontline nurses)*

(b) Paradigm shift: the tension between person-centered and task-centered care

Nurses may feel as though no critical thinking is expected of them and, at times, that they are merely service providers. An individual who delivers services does not need to think, does not have the capacity to change, and is obligated to merely execute orders:

*“I think the biggest factor related to this is that we, as employees, stopped being people and became numbers. What matters is having X staff working that day”. (FG1–PT-frontline nurses)*

Several responsibilities in the nursing job, and other duties unrelated to patient care, might divert a nurse’s energy. They can also increase workload stress, harm a nurse’s capacity to deliver person-centered care, and promote task-focused work practices:

*“The constant demand that has been happening... we are targeted, whether in terms of changing care and our demands, in terms of records, of what a nurse is supposed to do... attention to the needs of the patient and family members get called into question... and this wears professionals out”. (FG3-PT-nurse managers)*

Many institutional initiatives are not directly related to patient care or safety because of shortages in human resources, and as a result, participants expressed feelings of being overwhelmed:

*“But when we can’t show up, budgets get cut, and staffing is reduced. In the end, we become understaffed. Because the budget is not defined, let’s say. Unfortunately, this is also a reality. And so, that’s what creates all this pressure, I would say”. (FG6-CH-frontline nurses)*

Nurses appeared unable to control and regulate their workloads, torn between the care they would like to deliver and the care they were required to provide:

*“When there is no one else to replace me, I cannot be absent, because if I am absent, my patients will not have access to care”. (FG1-PT-frontline nurses)*

(c) Sudden changes in care practices due to the COVID-19 pandemic

The pandemic situation posed significant difficulties for nurses. Despite caring for patients with COVID-19 on a regular basis, many seemed lost in the jumble of shifting information, as if they were in the dark about what to do and where to receive help:

*“Everything was always changing, and it seemed that there was something different each day, and it was very difficult at first”. (FG2-PT-frontline nurses); “[There was] a lot of reorganization. Many sites started having 12-h shifts instead of the usual eight”. (FG1-PT-frontline nurses); “(…) the issue of unexpected mobility to go and reinforce the services in most need”. (FG4-PT-nurse managers)*

Amidst the chaotic scenario, some participants were sympathetic to their management and acknowledged that: *“They are doing the best they can” (FG1-PT-frontline nurses)*. Others, however, felt frustrated: *“They go to work, they are not prepared, they do not have time to get prepared, and of course, the first feeling is frustration”. (FG1-PT-frontline nurses)*

(d) Lack of shared work perspectives with hierarchical superiors

A lack of leadership may result in a breakdown in communication between nurses and management. Participants highlighted their management’s unwise support of organizational practices that compelled employees to be present at work:

*“Sometimes, the head nurses don’t help a person who comes into work despite having trouble doing so, because the managers pressure them to maintain the number of professionals present and consequently [staffing] ratios”. (FG2-PT-frontline nurses)*

They also stated their absence of trust and confidence in nurse managers—a previously identified barrier between nurses and management—as something preventing those managers from fully supporting, recognizing, sharing, and connecting with nurses:

*“So, sometimes, people find it hard to confide in us, because we’re managers, when in fact we’re just like them. But it is a fact that we’re managers and we’re [hierarchically] superior. It raises barriers”. (FG5-CH-nurse managers)*

(e) Financial burden of being absent from work

Presenteeism has been linked to personal and work-related issues. Participants clearly perceived and experienced significant financial consequences when absent from work, although the extent of the financial impact differed. Nurses’ dedication to their daily lives and issues involving their families had a constant influence on their presence at work, reflecting fears of losing income and facing further financial strain.

The sense of financial hardship started with the need to keep working because they were the family breadwinner:

*“The monetary part counts for a lot because a person who is used to [living on] a basic budget of one thousand euros, with family expenses, cannot afford to stay at home for a week to rest and recover—only if it’s really a life-or-death situation”. (FG2-PT-frontline nurses); “There’s also a lot of presenteeism linked to the financial side”. FG6-CH-frontline nurses)*

Moreover, if nurses take sick leave, they cannot even do part-time work:

*“There are people who cannot go to work because this is impactful from a financial point of view. Many nurses work part-time (…) and if they don’t work, if they are on sick leave, then they can’t work part-time either, and the impact is even greater and, therefore, this is also important”. (FG3-PT-nurse managers)*

(f) Misfit of human responses

Due to the stressful nature of their work, nurses developed different ways of dealing with the challenges of presenteeism imposed by their work on a daily basis. Individual actions that are viewed as misfits for suitable work relationships and a healthy organizational atmosphere were included in this theme. If nurses are to stay up to date, motivated, dedicated, and empowered, they need to work in an atmosphere that fosters ongoing professional growth. Some participants underlined the need to align nurses’ individual characteristics with particular clinical specialties, because a lack of nurse engagement results in less resilient individuals, with less energy (or vitality) for coping with their work’s demands, as the following quotes illustrate:

*“Forcing them to work in a place where the work is not what they like to do—it blows people up”; “Our way of being is a very important factor in how we react to a given situation”; “The internal variables [intrinsic to the person], such as motivation, interest, perhaps a certain resilience, that optimistic outlook that things will go well, these can be important factors that, when not present, lead to phenomena of this nature”. (FG4-PT-nurse managers) “I think it’s individual to each person and depends on the organizational culture, uh… I think it’s very personal”. (FG6-CH-frontline nurses)*

Participants were aware of some warning signs and symptoms of presenteeism; however, it is the normalization of this behavior that determines its presence in the workplace. They described having physical and mental problems, including fatigue, fluctuating moods, and mental stress:

*“(...) even if I’m physically tired, psychologically tired, or even depressed and I don’t feel able to come to work, that’s not quantifiable, it’s not taken seriously”. (FG6-CH-frontline nurse); “(...) do we have a good reason to miss work? Yes, we do! As long as it is fatigue or a depressive mood, it will always be justifiable. We are all tired!” (FG7-CH-frontline nurses)*

Although family support typically enhances work performance, commitment, and efforts to accept one’s job’s demands, a lack of this resource may affect the need for nurses′ presence at work. Support from colleagues may provide a stronger buffer against work’s strain than other sources of support (e.g., family) by increasing nurses’ sense of belonging and commitment. In this regard, participants said:

*“Often, people who are not very well, especially in terms of mental health, come to work to talk with their colleagues, to get a kind of moral support they wouldn’t necessarily get in their private life. I’ve often come across this with colleagues who, despite their inability—I mean—to be available for their work, came in because there was support from their colleagues. They could talk about their problems, etc., which they couldn’t do at home. So, I have the impression that there is also a lack of personal resources in their private lives and perhaps of someone in their private lives who’ll say, ‘No, you’re in no state to go to work.’ That’s it. (…) They come to work at all costs, in the end, to get help too”. (FG5-CH-nurse managers)*

Traditionally, the strongest justifications for absenteeism are the delays and deductions in pay and sometimes the total forfeiture of wages. Several participating nurses expressed concern about the effects of absenteeism, preferring “being presentee” even in bad conditions:

*“There is pressure (...) we know that if we are absent for more than a month, we will be fired. There was a political dynamic in my old job, where they instilled fear. So, often, employees have this fear of, ‘I have to work, I can’t afford to stop working (…), to stop getting a salary’, so they come to work... I even have stories of colleagues who came to work and vomited on the floor, and all because they knew they risked being [fired]”. (FG8-CH-nurse managers)*

## 4. Discussion

Presenteeism is viewed as a growing organizational problem [42]. In order to better understand this phenomenon, this study collected data about the concepts and experiences of presenteeism and its contributing factors from among frontline nurses and nurse managers in acute, primary, and long-term healthcare settings in Portugal and Switzerland [33]. The term *presenteeism* seemed to be new to several participants. Others defined the term as “going to work when unwell” but were unaware this was a well-known construct. This lack of awareness of the phenomenon did not mean they were not rapidly able to comprehend it. Some individuals appeared to favor doing everything they could to attend work when unwell, whereas others did not. Our findings corroborated a recent study [1] showing that occupational health and human resources management in nursing are dependent on knowledge about presenteeism. Nurses’ professional capacity to master health information is a key element of their core competencies, according to several publications [1,43]. As a result, nurses should have a high level of health literacy, be very concerned about remaining in good health, and be more successful at dealing with personal health issues [1]. They should know when to rest or request leave when they are unwell in order to recover more quickly [1]. Nevertheless, there is a significant disparity between nurses’ health knowledge and their harmful work behaviors (i.e., presenteeism). Although empirical research has shown that nurses are more health literate than the general population, they also have a higher rate of presenteeism than other professions [1,17,44]. Understanding why nurses continue working even when they are sick is, therefore, critical.

Some of the reasons given for presenteeism, such as a lack of knowledge, constraints on absenteeism, job resources, job demands, peer support, and health status, were found to be consistent with previous studies [12,45]. In addition to these known antecedents, other variables arose in our study, including a “paradigm shift: the tension between person-centered and task-centered care”; “sudden changes in care practices due to the COVID-19 pandemic”; and “misfit human responses”. 

Research shows that presenteeism has negative work consequences. It can lead to higher rates of short- and long-term absenteeism, lower productivity, and poorer health outcomes [46]. However, there is also evidence that presenteeism has some positive or useful attributes [47,48]. Some nurses in our study referred to how they had attended work when unwell and had received positive feedback for their conscientiousness, their commitment to the job and the organization, and for having spared their team members from having to replace them. Presenteeism is assumed as “a sustainable behavior when the presentee accomplishes work tasks within the boundaries of his or her reduced physical or mental resources” [49] (p. 247). Furthermore, presenteeism might function as a “survival strategy” for performing one’s duty or helping one’s peers; this view was also found by Giæver et al. [50]. Despite their poor health condition, nurses do not want to disappoint their colleagues. Rather than managerial pressure, the reason for presenteeism was attributed to the individual’s own choosing: a voluntary presentistic culture [7]. Hence, presenteeism can be unknowingly fostered because of the nursing role’s caring aspects, its reliance on teamwork, and the elevated feelings of loyalty [32]. According to Rainbow et al. [51], this culture of self-sacrifice encourages presenteeism and reporting for shifts when sick, and nurses expect their colleagues to do the same. As a result, Monneuse [52] wondered whether presenteeism was a choice or a constraint. He cited multiple studies suggesting that people would rather be around their colleagues than be alone and bored at home, especially if they are suffering from mood problems. Being absent, on the other hand, is not well received by management (involuntary presentistic culture), according to Ruhle and Süß [7], and may have severe consequences in the future, such as fewer opportunities for career progression.

In addition, and depending on the social security system, presenteeism may also alleviate the economic privations resulting from not working [48]. This is particularly prevalent in Portugal, where paid sick leave is limited or non-existent, and there is a mismatch between performance and reward. If they do not have paid sick leave, employees may be unable to afford the loss of revenue by remaining at home to recover [53]. In counterpoint, the Swiss healthcare system includes “strong ‘pull’ factors such as good working conditions with relatively high wages, good career pathways and opportunities for development and overall high job satisfaction” [54] (p. 161). 

Although presenteeism’s positive effects as a stabilizing component of team performance should be recognized, precautions should be put in place to avoid an insidious long-term deterioration in the health of substantial segments of the workforce [48]. This is particularly necessary in light of the COVID-19 pandemic. During sanitary crises, short staffing in certain organizations necessarily increases workloads and working hours, which might be aggravated by the need to cover for ill or vulnerable workers [55]. Recent studies showed that in the post-pandemic period, employees decided to engage in more extreme work behaviors in order to protect their employment and keep up with the demands of their occupations [56]. Our study indicated that the pandemic presented healthcare institutions with the challenging task of having to balance their need to stay operational with the need to maintain a healthy, satisfied, and motivated workforce. Although staff may tolerate such stresses in the short term, institutions may lose employees to more compassionate, people-centered organizations [57].

Our findings revealed that nurses go to work despite being physically or psychologically ill due to a variety of organizational and personal variables. Labor and organizational aspects such as the pressure of high workloads, a lack of support from supervisors or colleagues, and even the workplace environment may increase presenteeism. These findings support earlier studies on presenteeism in which some authors claimed that healthcare institutions encouraged this behavior [3,58]. Individual factors increasing presenteeism include feelings of abandoning colleagues in settings that are already understaffed, an inability to set personal boundaries, the need to earn a living, having a strong work ethic, and failing to recognize the severity of a disease [17,59].

From the perspective of hospitals or healthcare facilities, there seems to be a culture—particularly prevalent in Europe—of staff being expected to be present or seen [32]. In this respect, social interaction and workplace culture appear to have a considerable impact on nurses’ individual and collective behaviors and perceptions. Despite their explicit criticism of task-focused work organization, nurses in the present study felt that their delivery of person-centered care was restrained and that they responded in ways that fostered a focus on tasks. As a result, the components of healthcare that organizations see as having little economic value, such as the relational aspects of care, were at risk of being neglected and overlooked [60].

Our study underlined that a lack of support from hierarchical superiors and institutions’ disinterest in their staff were relevant factors contributing to presenteeism. A previous meta-analysis showed negative associations between presenteeism and co-worker support, interpersonal relationships, and supervisory support [45]. Despite this, positive support and relationships indirectly increased presenteeism via a positive impact on job satisfaction [45]. Europe’s Latin-language nations, such as Portugal, tend to be characterized by charismatic leaders and supervision that encourages a potentially collaborative cultural dimension [61]. On the other hand, there is a significant level of distrust between managers and employees in non-Latin nations [61,62]. In very multicultural societies [63]—the environment found in Switzerland—trust between these groups is considered to be more difficult to establish since people do not always share the mental models that facilitate mutual understanding, and they are thus more prone to interpreting situational events and management methods differently [63].

Healthcare facilities with significant corporate cultural barriers strengthen professional norms against taking sick leave, which might unintentionally encourage presenteeism [19]. This evidence is similar to our findings, as some participating nurse managers recognized presenteeism as indispensable for functioning teams.

Interestingly, financial needs acted as a contributing factor to presenteeism among frontline nurses. Previous research found that nurses with significant personal financial needs were at a higher risk of presenteeism [1]. This fact may explain why, in the face of physical discomfort, persons with lower incomes are more inclined to choose presenteeism than absenteeism. Salary disparities between head nurses and their subordinates may also influence these decisions; head nurses frequently earn more than regular nurses, which may cause them to undervalue the impact of financial obligations on nurse presenteeism [1].

In our findings, misfitting responses were a relevant factor of nurse presenteeism. Misfit is typically assumed as an extreme of the fit spectrum, implying discomfort or incompatibility. Scholars have argued that more attention should be devoted to the misfit situation to better understand how people deal with it [64,65]. Misfits, according to these researchers, are somewhat adaptable and may be influenced by employee thoughts and behaviors. According to existing studies, the option to remain misfit rather than leaving for other opportunities is a difficult one. Evidence suggests, however, that highly responsible employees are more likely to be presentees [1]. Individuals with high levels of responsibility are more inclined to accomplish tasks on their own rather than seek assistance from others [66,67]. Physical or mental discomfort does not discourage them [1]. Furthermore, highly conscientious people are more concerned about how their absence would affect both them as individuals and their organization [1,16]. They fear, for example, that their absence will harm their image as leaders and cause problems with shift scheduling for managers and the organization. 

Several participants attempted to address the underlying causes of their perceived person–environment misfit. They worked to re-establish a sense of fit by altering either the surroundings or themselves. As a result, even if they appeared to accept their misfit, they were still actively engaged in efforts to limit its effects and modify either themselves or their environment. According to a study by Pit and Hansen [41], presenteeism is promoted by a lack of personal health resources aligned with a poor work–life balance and negative organizational factors. Our findings supported this evidence and suggested that some workplace cultures inadvertently foster and normalize presenteeism [68]. This might be why nurses have difficulty balancing their work with personal and family issues and have very limited time to care for themselves, with all the negative consequences on their health and productivity that might ensue. 

Despite the particularities of Portugal and Switzerland’s cultures and working environments, nurses’ perceptions of the concept and causes of presenteeism were quite similar. According to Maaravi et al. [69], national features and culture have a key impact on how nurses respond to health problems and, as a result, presenteeism. In both countries, great importance was given to a job well done and to the shared values of hard work and perseverance; however, there was also a shared perception of the legitimacy of absences across both cultures, and these may all play roles in understanding decisions to work when unwell [9,10,70].

### 4.1. Strengths and Limitations

The diversity of individuals who participated in the FG discussions is one of the study’s strengths. There was a mixture of different types of nurses from various clinical settings and with different perceptions of the phenomenon under study. Another strength was that our research was conducted in two different cultural settings. We used a similar semi-structured interview guide for both countries’ FGs, covering the same basic structure and topics; however, we also considered national contexts and cultural specificities to clarify or slightly reformulate some questions. Interviews were conducted in participants’ native languages (Portuguese and French). Moderators used a constructivist approach when asking questions and let participants steer the conversation based on their personal experiences.

This study also had some potential methodological limitations. First, our approach to participant recruitment might have been susceptible to selection bias (e.g., nurses with and without previous experiences of presenteeism). For example, presentees focused more on their own conduct and non-presentees focused more on a fictitious decision-making process; however, we cannot rule out the possibility that the decision processes changed between the two groups [71]. This should be considered when interpreting the results.

Second, the study’s cross-sectional design might be viewed as a limitation. We thus recommend the development of multisite, longitudinal research in a variety of work settings. Third, new factors linked to workers’ health (such as mental health, chronic/acute sickness, or occupational diseases) might be added to future studies to examine their possible links and negative repercussions for nurses. This might require employing a larger sample size. Lastly, this study was conducted at the onset of the COVID-19 pandemic. Thus, the novelty and associated uncertainty of the entire COVID-19 situation may have influenced the results. 

### 4.2. Study Implications

The development of consistent methods to prevent and minimize presenteeism among nurses will benefit from a comprehensive understanding of the phenomenon’s contributing factors. According to Hemp [72], raising awareness, identifying concerns, and education should be the focus of workplace interventions to reduce the impact of presenteeism and promote decent work for all [73]. Nurse leaders and human resource managers should pay attention to the amount of job stress their staff are experiencing [74], as this has been linked to presenteeism. They should train staff and create policies to change their behaviors, such as promoting well-being and work–life balance by having more flexible schedules, maintaining constant feedback possibilities to combat uncertainties, and enhancing communication strategies, such as via continuing professional education [75].

Managers should be aware of and trained to recognize the early warning signs of common physical, emotional, and mental health issues in the workplace, so that they can act as soon as possible and communicate supportively with employees who are having health problems. Healthcare facilities should conduct regular evaluations of their workers’ well-being to reveal their true health status and implement worksite wellness programs to help health professionals improve their physical and mental health. Effective interventions, such as occupational health strategies and counselling, might help nurses improve the professional quality of their care and boost their occupational health in terms of physical activity, stress reduction, sleep hygiene, work–life balance, and the physical demands of their job [41]. Moreover, to guarantee that both staff and patients stay safe, best-practice disaster preparedness and management strategies must take into consideration healthcare professional presenteeism and address its drivers [31,55,76]. Attending to nurses’ needs will not only strengthen their well-being and work ethic but will also have a positive impact on teamwork and the quality of care delivered to patients [77]. Decision-makers, managers, and leaders should establish healthcare organization guidelines and interventions to better equip nurses for the post-pandemic working environment [9,78]. 

A better understanding of presenteeism among the nurses in both countries could be facilitated by the development of strategies to combat it presented in nursing students’ curricula. Assertiveness training, the promotion of self-esteem, resilience skills, and ergonomic training should be applied via simulation-based training programs that help students transfer their learning outcomes to clinical practice. This might also improve self-confidence and team performance. Closer collaboration between higher education establishments and institutional clinical settings has been advocated to improve the translation of learning results from simulation to clinical practice [79].

## 5. Conclusions

This qualitative study generated valuable, in-depth knowledge about the concepts and causes of presenteeism and instructive input on the subject for a broad audience of nurse managers and healthcare leaders. According to our thematic analysis, numerous elements go towards explaining presenteeism. They are closely connected with institutions placing demands on their staff to attend work, personal restrictions and commitments that staff place on themselves, and organizational environments. There are a variety of reasons why nurses report for work despite their inability to work effectively or efficiently. When it came to presenteeism, we found evidence that staff members’ individual perspectives about how to deal with their health and sickness generally played a pivotal role. In addition, presenteeism was influenced by organizational issues, such as a lack of any replacements when an employee was off sick or a professional nursing culture where taking sick leave was seen as a weakness. Finally, contextual variables, such as a lack of sufficient legal protection against dismissal due to illness, were key drivers of presenteeism and reflected cultural variations between the countries. These characteristics must be considered when devising interventions to prevent presenteeism, increase the quality and safety of patient care, promote staff well-being, and to maximize overall healthcare performance. To turn presenteeism into a positive historical experience, the nurses who strive to provide their patients with optimal care must proactively take care of themselves and their colleagues in the same attentive manner.

## Figures and Tables

**Table 1:** Six steps for the thematic analysis [39].

1. Data familiarization: transcription, reading, re-reading, and taking notes.
2. Initial code generation.
3. Searching for themes by sorting codes into prospective themes and concentrating all the required data for each potential theme.
4. Reviewing themes: creating a thematic “map”.
5. Theme definition and naming.
6. Producing the report.

**Table 2:** Sociodemographic and professional characteristics of the participants (*n* = 55).

	Focus Groups (FG1–FG4) Portugal	Focus Groups (FG5–FG8) Switzerland
**Number of participants**	39	16
**Work function**		
Frontline nurses	20	8
Nurse managers	19	8
**Sex**		
Female	34	14
Male	5	2
**Educational level**		
Bachelor’s degree	20	6
Master’s degree	19	9
PhD degree	0	0
**Age**—mean (SD)	43.86 (8.96)	46.19 (12.43)
**Years of experience in healthcare**—mean (SD)	17.80 (8.41)	20.75 (11.64)
**Healthcare setting**		
Acute	39	0
Primary	0	10
Long-term	0	6

## Data Availability

The data presented in this study are available on request from the corresponding author. The data are not publicly available due to containing information that could compromise the privacy of research participants.
